# Gallstone Disease Increases the Risk of Kidney Stone Disease: A Cross-Sectional and Longitudinal Cohort Study

**DOI:** 10.7150/ijms.128864

**Published:** 2026-04-08

**Authors:** Yu-Ting Lin, Jia-In Lee, Szu-Chia Chen, Shu-Pin Huang, Jiun-Hung Geng

**Affiliations:** 1 Post Graduate Year (PGY) Training, MacKay Memorial Hospital, Taipei 10449, Taiwan.; 2 Department of Psychiatry, Kaohsiung Medical University Hospital, Kaohsiung Medical University, Kaohsiung 807378, Taiwan.; 3 Department of Internal Medicine, Kaohsiung Municipal Siaogang Hospital, Kaohsiung Medical University, Kaohsiung 812015, Taiwan.; 4 Department of Internal Medicine, Division of Nephrology, Kaohsiung Medical University Hospital, Kaohsiung Medical University, Kaohsiung 807378, Taiwan.; 5 Faculty of Medicine, College of Medicine, Kaohsiung Medical University, Kaohsiung 807378, Taiwan.; 6 Center for Big Data Research, Kaohsiung Medical University, Kaohsiung 807378, Taiwan.; 7 Graduate Institute of Clinical Medicine, College of Medicine, Kaohsiung Medical University, Kaohsiung 807378, Taiwan.; 8 Department of Urology, Kaohsiung Medical University Hospital, Kaohsiung Medical University, Kaohsiung 807378, Taiwan.; 9 Department of Urology, School of Medicine, College of Medicine, Kaohsiung Medical University 807378, Kaohsiung, Taiwan.; 10 Department of Urology, Kaohsiung Municipal Siaogang Hospital, Kaohsiung 812015, Taiwan.

**Keywords:** gallbladder stone, gallstone disease, kidney stone disease, urolithiasis, cohort study

## Abstract

**Background:**

Gallstone disease (GSD) and kidney stone disease (KSD) may share metabolic pathways, however their association remains unclear. This study aimed to explore their relationship using cross-sectional and longitudinal analyses.

**Methods:**

The cross-sectional analysis included 121,550 participants in the Taiwan Biobank, and the association between self-reported GSD and KSD was examined using logistic regression. The longitudinal analysis included 25,208 participants without prior KSD, and the risk of incident KSD was assessed using Kaplan-Meier survival analysis and Cox proportional hazards models, adjusting for demographics, metabolic factors, lifestyle factors, and laboratory data.

**Results:**

In the cross-sectional cohort, the prevalence of KSD was higher in the participants with GSD (11% vs. 6%, p < 0.001). In adjusted analysis, GSD was associated with a 51% increased risk of KSD (odds ratio: 1.51, 95% CI: 1.38-1.65, p < 0.001). In the longitudinal analysis, the participants with GSD had a higher incidence of KSD (5% vs. 2%, p < 0.001), with an adjusted hazard ratio of 1.80 (95% CI: 1.36-2.38, p < 0.001). Subgroup analyses showed a significant interaction by sex but not by age, indicating a stronger association in women.

**Conclusion:**

Our results showed that GSD was an independent risk factor for KSD, suggesting shared metabolic mechanisms. Future studies should explore preventive strategies targeting the pathways underlying these mechanisms.

## Introduction

Gallstone disease (GSD) is characterized by the presence of gallstones composed primarily of cholesterol, bile salts, and calcium in the gallbladder. It is a common biliary system disorder with a global prevalence of approximately 6.1%, including 5.4% in men and 7.6% in women 1, and a higher prevalence of 10.7% in Taiwan 2. Older age and female sex are important risk factors for gallstones.

Gallstones can be broadly classified into three categories: cholesterol stones, black pigment stones, and brown pigment stones. If the flow of bile through the biliary system is obstructed by a gallstone, it can affect any related organ and potentially cause cholecystitis, cholangitis, hepatitis, pancreatitis and sepsis. These diseases lead to an increase in emergency department visits and hospitalization rates, resulting in higher healthcare burden and costs.

Kidney stone disease (KSD) is a common disorder of the urinary system characterized by the presence of kidney stones typically composed of uric acid, calcium oxalate, or calcium phosphate. KSD is highly prevalent worldwide, with rates ranging from 7% to 13% in North America, 5% to 9% in Europe, and 1% to 5% in Asia 3. In Taiwan, the reported prevalence is 7.38%, with a 5-year recurrence rate of 34.71% 4. KSD is associated with high healthcare cost due to the high incidence of new and recurrent stones, and the condition is linked to considerable acute and chronic morbidity, including severe pain, urinary tract infections, and chronic kidney disease.

While KSD and GSD differ in anatomical location and composition, emerging evidence suggests that they may share common risk factors and pathological mechanisms. The pathophysiology of gallstone and kidney stone formation involves complex interactions between genetic, metabolic, and environmental factors. In addition to dietary habits, conditions such as obesity, diabetes and metabolic syndrome are well-documented contributors to both types of stones.

Understanding the relationship between gallstones and kidney stones is clinically significant, as it may provide insights into shared preventive strategies and improve patient outcomes. In this study, we used both a cross-sectional and longitudinal cohort study design to explore the potential link between GSD and KSD in a large community-based cohort in Taiwan.

## Materials and Methods

### Data source and study population

**Figure 1:**
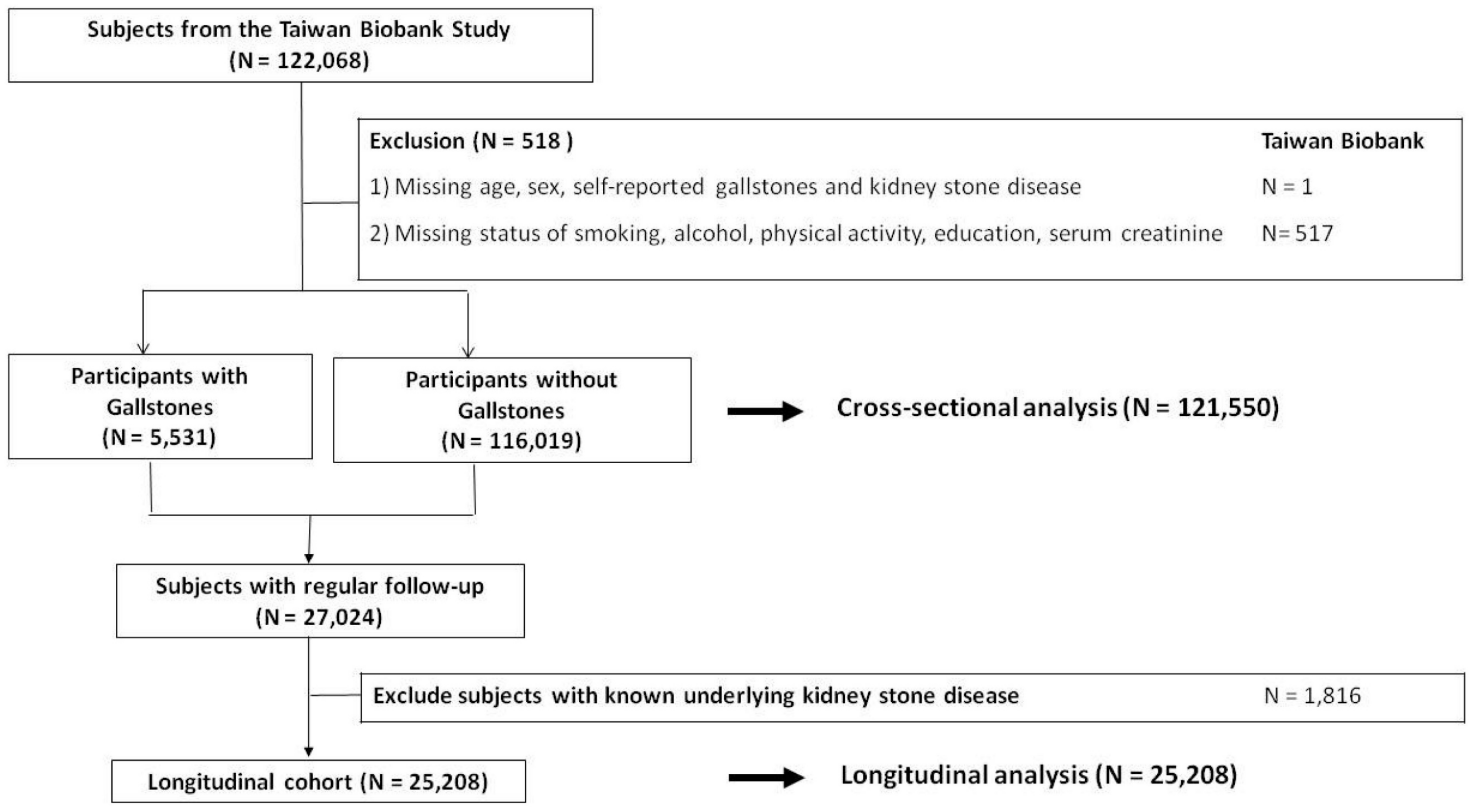
Study participants were classified by the presence of gallstones.

The study cohort included participants in the Taiwan Biobank (TWB), a large community-based research project that has been described in detail in previous studies 5-9. In brief, the TWB was established in 2008 and recruits cancer-free individuals aged 30 to 70 years from 29 centers around Taiwan. In the cross-sectional part of this study, we included 121,550 participants with complete baseline data (518 of the total 122,068 participants in the TWB were excluded due to missing data), as shown in **Figure 1**.

In the longitudinal cohort, we identified 27,024 of the 121,550 participants who had regular follow-up data. We then excluded 1,816 of these participants who had a known history of KSD, and included the remaining 25,208 participants (Figure 1). From 2008 to 2019, all of the participants completed a physical examination, biospecimen collection, and a questionnaire every 2 to 4 years. In addition, all of the participants provided written informed consent, and the study adhered to the Declaration of Helsinki. The Institutional Review Board of Kaohsiung Medical University Hospital approved the study (KMUHIRB-E(I)-20210058).

### Self-Reported GSD

The presence of GSD was collected by asking the following questions during standardized interviews: “Have you ever had GSD?” and, if so, “When were you diagnosed?” The same questions were asked again in follow-up interviews every 2 to 4 years. In the longitudinal analysis, GSD status was defined at baseline based on self-reported history. Participants were classified according to their baseline GSD status and were not reclassified during follow-up, even if new gallstones were reported. This approach was adopted to preserve a fixed baseline exposure definition and to avoid potential immortal time bias that could arise from time-dependent exposure misclassification 10.

### Self-Reported KSD

Similarly, the presence of KSD was obtained by asking, “Have you ever had KSD?” and, if so, “When were you diagnosed?” These questions were also repeated in follow-up interviews conducted every 2 to 4 years.

### Study Outcomes

The participants were first classified into gallstone and no gallstone groups. In the cross-sectional cohort, we evaluated the association between GSD and the prevalence of KSD. In the longitudinal cohort, the primary endpoint was the development of KSD. None of these participants had a history of KSD at baseline. We analyzed the relationship between GSD and the subsequent occurrence of KSD to explore potential associations between the two conditions. We also performed subgroup analyses considering that age and sex are important risk factors with the following four groups: Age ≤ 50 years, Age > 50 years, Men, and Women.

### Statistical Analyses

Clinical characteristics were expressed as percentages for categorical variables and as mean ± standard deviation for continuous variables. The Pearson χ² test was used to evaluate differences in categorical variables, while an independent t-test was used to compare continuous variables between the participants with and without gallstones.

In the cross-sectional cohort, logistic regression was performed to examine the association between GSD and the prevalence of KSD, both before and after adjusting for potential epidemiologic factors (age, sex, body mass index, waist circumference, smoking status, alcohol consumption, physical activity, and education level) as well as laboratory parameters (serum creatinine, fasting glucose, total cholesterol, triglycerides, and uric acid).

For the longitudinal cohort, Kaplan-Meier analysis and the log-rank test were used to estimate and compare cumulative event-free survival rates. Event-free survival time was defined as the period from baseline to the primary endpoint or the last recorded follow-up date. Participants who either died or were lost to follow-up were censored at the date of their last examination.

All statistical analyses were conducted using SPSS version 20.0 (IBM Corp, Armonk, NY, USA) and R version 3.6.2 (R Foundation for Statistical Computing, Vienna, Austria). R was used for data cleaning and preparation, whereas SPSS was used for baseline comparisons, logistic regression analyses, survival analyses, and Kaplan-Meier curve generation. A two-sided p-value < 0.05 was considered statistically significant.

## Results

### Clinical Characteristics of the Study Participants

There were significant differences in baseline characteristics between those with and without gallstones. Among the 121,550 participants, 5,531 (4.6%) were diagnosed with GSD (Table 1). The participants with gallstones were generally older than those without and had higher rates of comorbidities including hypertension, diabetes mellitus, dyslipidemia, gout, irritable bowel disease, and chronic kidney disease (Table 1). In addition, the participants with gallstones had significantly higher body mass index and waist circumference, both of which are established metabolic risk indicators (Table 1). There were also differences in lifestyle factors between the two groups, and the participants with gallstones had a slightly higher proportion of ever-smokers (Table 1). Moreover, the participants with gallstones had significantly higher fasting glucose and triglyceride levels (Table 1), suggesting a possible link between GSD and metabolic problems.

### GSD was Associated with a Higher Risk of KSD in the Cross-sectional Cohort

The association between GSD and KSD was analyzed using both unadjusted and adjusted models. Among the 121,550 participants, the prevalence of KSD was significantly higher in the those with gallstones (11% vs. 6%, p < 0.001) (Table 2). In univariate binary regression analysis without adjustment, GSD was associated with a 1.92-fold increased risk of KSD (odds ratio [OR]: 1.92, 95% confidence interval [CI]: 1.76-2.09, p < 0.001), and the risk remained statistically significant even after adjusting for multiple confounders (adjusted OR: 1.51, 95% CI: 1.38-1.65, p < 0.001) (Table 2). Subgroup analyses showed that the association between GSD and KSD was present across all age and sex categories. In participants aged ≤ 50 years and > 50 years, the adjusted ORs were 1.40 (adjusted OR: 1.40, 95% CI: 1.15-1.70, p = 0.001) and 1.54 (adjusted OR: 1.54, 95% CI: 1.39-1.70, p < 0.001), respectively. Similarly, the adjusted ORs were 1.49 (OR: 1.49, 95% CI: 1.32-1.68, p < 0.001) in men and 1.53 (OR: 1.53, 95% CI: 1.34-1.76, p < 0.001) in women (Table 2). However, formal interaction testing revealed no statistically significant interaction by age or sex (both p for interaction > 0.05), indicating that the differences in subgroup estimates likely reflect random variation rather than true effect modification.

### Clinical Characteristics of the Longitudinal Cohort

Among 25,208 participants without prior KSD, 1,144 (4.5%) had gallstones at baseline (Table 3). Participants with gallstones were older and exhibited less favorable metabolic profiles, including higher body mass index and waist circumference (all p < 0.001). They also had a greater prevalence of hypertension, diabetes mellitus, and dyslipidemia compared with those without gallstones (all p < 0.001). Laboratory findings were consistent with this pattern, as the gallstone group had higher fasting glucose, triglyceride, and uric acid levels. Although follow-up duration was slightly shorter in participants with gallstones, the absolute difference was minimal.

### GSD Was Associated with a Higher Risk of KSD in the Longitudinal Cohort

The longitudinal analysis revealed a significant association between GSD and the incidence of KSD (Table 4). The participants with gallstones had a higher incidence of KSD (5% vs. 2%), with an unadjusted hazard ratio (HR) of 2.14 (95% CI: 1.62-2.82, p < 0.001). After adjusting for multiple confounders including age, sex, metabolic factors, lifestyle factors, comorbidities and laboratory data, the association remained significant** (**adjusted HR: 1.80, 95% CI: 1.36-2.38, p < 0.001).

Subgroup analyses showed that the association between GSD and incident KSD was present across age groups, with adjusted HRs of 2.06 (HR: 2.06, 95% CI: 1.23-3.43, p = 0.006) in participants aged ≤ 50 years and 1.73 (HR: 1.73, 95% CI: 1.24-2.41, p = 0.001) in those aged > 50 years. However, formal interaction testing showed no statistically significant interaction by age (P for interaction = 0.416).

In contrast, sex-stratified analyses suggested heterogeneity of effect. The adjusted HR was 1.37 (adjusted HR: 1.37, 95% CI: 0.91-2.07, p = 0.135) in men and 2.44 (adjusted HR: 2.44, 95% CI: 1.66-3.58, p < 0.001) in women, and the interaction between GSD and sex was statistically significant (p for interaction = 0.043), indicating a stronger association in women.

**Figure 2:**
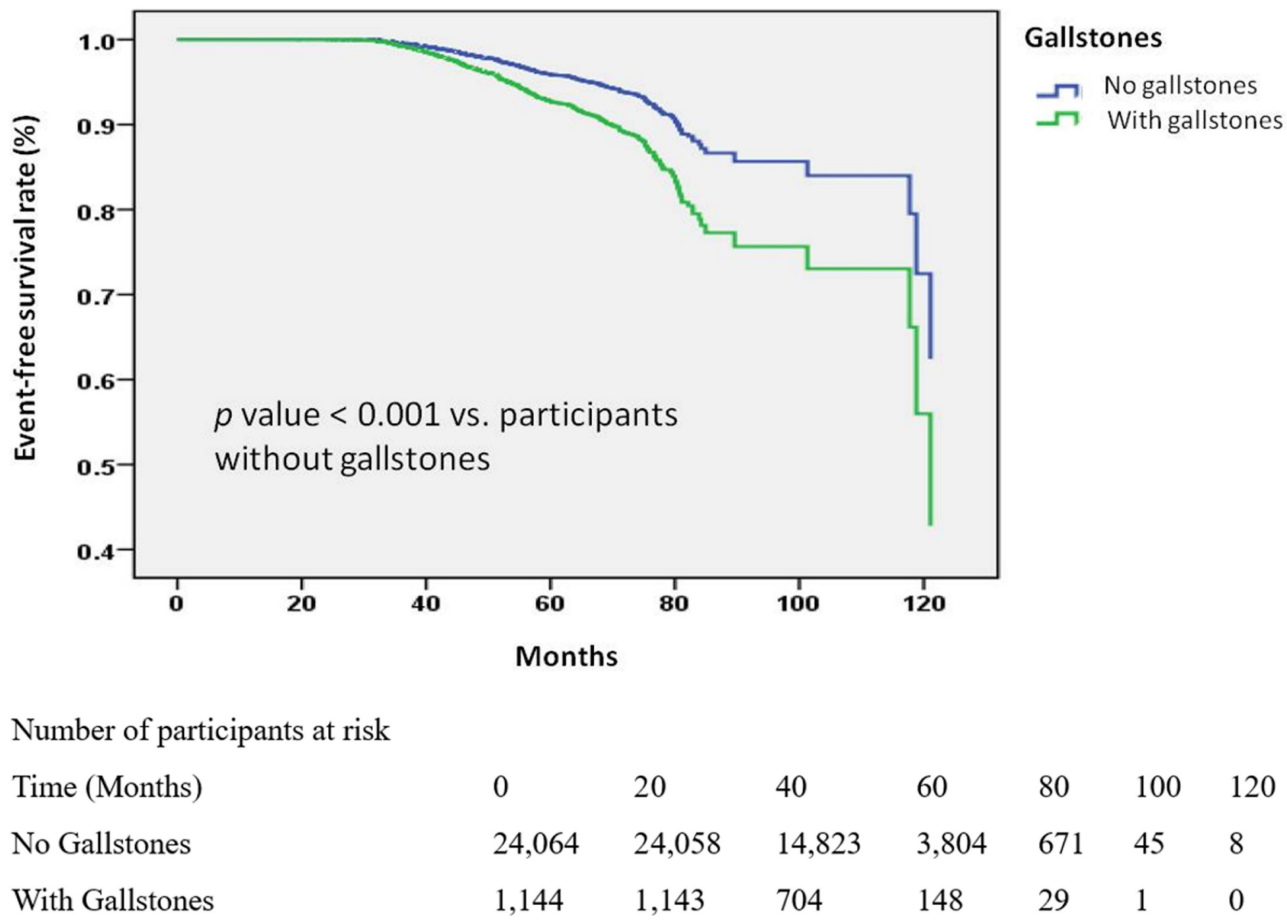
**Kaplan-Meier Curves for the Development of Kidney stone disease (KSD) Stratified by Gallstone Status.** Kaplan-Meier survival curves depicting the time to KSD development in the participants with and without gallstones. The participants without gallstones had a longer time to the onset of KSD compared to those with gallstones. The analysis included 25,208 participants with available follow-up data.

Kaplan-Meier analysis (Figure 2) provided consistent visual confirmation, showing a higher cumulative incidence of KSD among participants with gallstones compared with those without (log-rank p < 0.001).

## Discussion

In this nationwide population-based study, GSD was independently associated with both prevalent and incident KSD. No effect modification by age was observed, whereas a significant interaction by sex was identified in the longitudinal analysis, with a stronger association in women. To our knowledge, this is among the largest population-based studies in Asia examining the association between GSD and KSD.

Several previous studies from Europe and the United States support our findings. In a cross-sectional study, Akoudad et al. identified an association between KSD and GSD among White (OR 1.46, p < 0.001) and African-American (OR 3.01, p < 0.001) subjects 11. Similarly, in another cross-sectional and prospective study, Taylor et al. demonstrated that both stone diseases were independently associated with older women, young women, and men 12. Moreover, in a cross-sectional study of patients with inflammatory bowel disease, Fagagnini et al. found that the presence of gallstones significantly increased the risk of kidney stones (OR 4.87, p < 0.001) 13. However, these studies did not adjust for potential risk factors such as BMI, waist circumference, smoking, and physical activity. In contrast, our study included over 100,000 participants, of whom more than 20,000 had sufficient follow-up data, and we collected comprehensive data on age, sex, BMI, waist circumference, smoking status, physical activity, educational background, comorbidities, and laboratory parameters, allowing evaluation of the temporal sequence between GSD and subsequent KSD occurrence.

In addition to the studies from Europe and the United States supporting our findings, several studies from Asia have also reported similar results. In a large-scale nationwide cohort study using the Taiwan National Health Insurance Research Database (NHIRD), Li et al. demonstrated that GSD significantly increased the risk of developing KSD, with an adjusted HR of 1.68, suggesting a strong association between the two conditions 14. Similarly, in a Korean longitudinal follow-up study using national health insurance data, Kim et al. identified a bidirectional relationship between GSD and KSD, reporting an HR of 1.93 for kidney stones in gallstone patients and 1.97 for gallstones in kidney stone patients 15. Furthermore, a recent multicenter study in China also confirmed this association, highlighting that metabolic and inflammatory pathways may contribute to the co-development of these diseases 16. The consistency of findings across diverse populations suggests that this association is not region-specific, but rather a fundamental epidemiological link that warrants further mechanistic investigations.

Although many studies support a link between GSD and KSD, some have reported different results. A recent study from South Korea on patients with intestinal Behçet's disease found no significant correlation between the occurrence of gallstones and kidney stones 17. In another study, Song et al. identified risk factors for each condition, such as prolonged disease duration, frequent hospitalizations, and prior intestinal Behçet's disease-related surgery, but did not establish a direct link between GSD and KSD 17. In contrast, our cross-sectional and longitudinal cohort studies both demonstrated a clear association between GSD and KSD. Furthermore, most of our participants were healthy, cancer-free, and aged 30 to 70 years rather than a population with specific underlying diseases, making our findings more generalizable to the broader population.

The potential mechanisms linking GSD and KSD are complex and multifactorial. Several studies have suggested that metabolic syndrome, insulin resistance, and shared risk factors such as obesity, hypertension, and dyslipidemia play crucial roles 11, 12, 18. Insulin resistance may lead to increased hepatic cholesterol secretion and consequently promote gallstone formation, while simultaneously altering renal acid-base metabolism and consequently lowering urinary pH and increasing the risk of uric acid stone formation 13. Furthermore, systemic inflammation, which is often associated with metabolic syndrome, may contribute to both gallstone and kidney stone formation through oxidative stress and chronic inflammatory responses 15. In addition, alterations in gut microbiota and bile acid metabolism can influence intestinal oxalate absorption, potentially increasing the risk of calcium oxalate kidney stones 14. Some studies have also proposed that dysfunction of epithelial transporters in both the gallbladder and renal tubules may create an environment conducive to stone formation in both the gallbladder and kidneys 16. Overall, these mechanisms suggest that GSD and KSD share common metabolic and physiological pathways, supporting the association between GSD and an increased risk of developing KSD.

In our subgroup analysis, a stronger association was observed in women but not in men. This sex-specific difference may relate to hormonal influences on bile composition, lipid metabolism, and urinary chemistry, all of which have been implicated in both gallstone and kidney stone formation 19-21. Estrogen exposure is known to promote cholesterol supersaturation in bile, while also affecting urinary citrate excretion and acid-base balance. Nevertheless, this finding should be interpreted cautiously because subgroup analyses may be underpowered, and confirmation in independent cohorts is warranted.

Although the associations observed in our study were statistically significant, the magnitude of effect (adjusted OR 1.51 in the cross-sectional analysis and adjusted HR 1.80 in the longitudinal analysis) indicates a moderate relative increase in risk rather than a large effect size. In epidemiologic research, relative risks in the range of 1.3-2.0 are generally considered modest but potentially meaningful, particularly when the exposure and outcome are both common in the population 3,4. Therefore, GSD should be interpreted as one component within a broader metabolic susceptibility profile rather than as a dominant or isolated determinant of KSD. Given the high prevalence of both gallstone disease and kidney stone disease worldwide 1,3,4, even moderate associations may have implications at the population level, although the absolute risk increase at the individual level remains limited.

Several limitations should be acknowledged. First, both GSD and KSD were ascertained through self-reported questionnaires rather than imaging confirmation or medical record validation, which may have introduced misclassification, particularly for asymptomatic or undiagnosed cases. Because exposure and outcome were assessed independently using standardized questionnaires, any misclassification is likely to be nondifferential and would be expected to bias effect estimates toward the null 22-24. Second, surveillance bias cannot be excluded. Individuals with gallstone disease may have more frequent healthcare encounters or undergo abdominal imaging, increasing the likelihood of incidental kidney stone detection. Differential diagnostic intensity could partially inflate the observed association, particularly in cross-sectional analyses. Third, although we adjusted for a comprehensive set of demographic, metabolic, lifestyle, and laboratory variables, some metabolic conditions (diabetes, dyslipidemia, and gout) may lie on the potential causal pathway between GSD and KSD rather than acting purely as confounders. Adjustment for such intermediate variables could introduce overadjustment bias and attenuate the magnitude of the association. Conversely, residual confounding from unmeasured factors, including dietary intake, fluid consumption, and genetic predisposition, cannot be fully excluded 25. Fourth, although our cross-sectional analysis identified a significant association between GSD and KSD, causality cannot be established due to the study design. Nonetheless, the longitudinal cohort analysis provided supportive evidence consistent with a temporal sequence between GSD and subsequent KSD development. Lastly, the follow-up period may not have been sufficient to identify all incident cases of KSD, potentially underestimating the true association. Future prospective studies with objective diagnostic measures, longer follow-up duration, and more diverse populations are warranted to further validate our findings and explore underlying mechanisms.

## Conclusion

Our results showed that GSD was an independent risk factor for KSD, suggesting shared metabolic mechanisms. Future studies should explore preventive strategies targeting the pathways underlying these mechanisms.

## Figures and Tables

**Table 1:** Baseline Clinical Characteristics of the Study Participants Stratified by the Presence of Gallstones (N = 121,550)

Characteristics	Total (N = 121,550)	Gallstones (N = 5,531)	No Gallstones (N = 116,019)	*p* value
**Demographic data**				
Age, yr	50±11	55±10	50±11	< 0.001
Men, n (%)	43,679 (36)	2,078 (38)	41,601 (36)	< 0.001
Body mass index, kg/m^2^	24±4	25±4	24±4	< 0.001
Waist circumference, cm	83±10	86±10	83±10	< 0.001
Smoke, ever, n (%)	33,134 (27)	1,628 (29)	31,506 (27)	< 0.001
Alcohol status, ever, n (%)	10,351 (9)	484 (9)	9,867 (9)	0.523
Physical activity, yes, n (%)	49,286 (41)	2,531 (46)	46,7555 (40)	< 0.001
Educational status ≥ College, n (%)	70,447 (58)	2,946 (53)	67,501 (58)	< 0.001
**Comorbidity**				
Hypertension, n (%)	14,881 (12)	1,199 (22)	13,682 (12)	< 0.001
Diabetes mellitus, n (%)	6,274 (5)	543 (10)	5,731 (5)	< 0.001
Dyslipidemia, n (%)	9,038 (7)	751 (14)	8,287 (7)	< 0.001
Gout, n (%)	4,675 (4)	296 (5)	4,379 (4)	< 0.001
Irritable bowel syndrome, n (%)	3,024 (3)	216 (4)	2,808 (2)	< 0.001
Chronic kidney disease, n (%)	1,385 (1)	103 (2)	1,282 (1)	< 0.001
**Laboratory data**				
Creatinine, mg/dL	0.7±0.3	0.7±0.4	0.7±0.3	< 0.001
Fasting glucose, mg/dl	96±20	99±23	96±21	< 0.001
Total cholesterol, mg/dL	196±36	195±36	196±36	0.073
Triglyceride, mg/dL	116±94	123±79	115±95	< 0.001
Uric acid, mg/dL	5.4±1.4	5.5±1.3	5.4±1.4	< 0.001

Continuous variables are presented as mean ± standard deviation

**Table 2:** Association Between GSD and KSD: Prevalence and Adjusted Odds Ratios Stratified by Age and Sex (N = 121,550)

Variables	No. of kidney stone cases (%)	Number at risk	Unadjusted odds ratio (95% CI)	*p* value	Adjusted odds ratio (95% CI)*	*p* value**
**All**						
Gallstones, no	7,118 (6)	116,019	1.00 (Reference)	-	1.00 (Reference)	-
Gallstones, yes	616 (11)	5,531	1.92 (1.76 to 2.09)	< 0.001	1.51 (1.38 to 1.65)	< 0.001
**Age ≦ 50 years old**						
Gallstones, no	2,543 (4)	58,102	1.00 (Reference)	-	1.00 (Reference)	-
Gallstones, yes	124 (7)	1,668	1.76 (1.46 to 2.12)	< 0.001	1.40 (1.15 to 1.70)	0.001
**Age > 50 years old**						
Gallstones, no	4,575 (8)	57,917	1.00 (Reference)	-	1.00 (Reference)	-
Gallstones, yes	492 (13)	3,863	1.70 (1.54 to 1.88)	< 0.001	1.54 (1.39 to 1.70)	< 0.001
**Men**						
Gallstones, no	4,290 (10)	41,601	1.00 (Reference)	-	1.00 (Reference)	-
Gallstones, yes	367 (18)	2,078	1.87 (1.66 to 2.10)	< 0.001	1.49 (1.32 to 1.68)	< 0.001
**Women**						
Gallstones, no	2,828 (4)	74,418	1.00 (Reference)	-	1.00 (Reference)	-
Gallstones, yes	249 (7)	3,453	1.97 (1.72 to 2.25)	< 0.001	1.53 (1.34 to 1.76)	< 0.001

GSD, gallstone disease; KSD, kidney stone disease; CI, confidence interval. *Multivariable analysis adjusted for age, sex, body mass index, waist circumference, smoking status, physical activity, educational status, presence of hypertension, diabetes mellitus, dyslipidemia, gout, irritable bowel syndrome, chronic kidney disease, serum creatinine, fasting glucose, triglycerides, and uric acid. **p for interaction was obtained by adding multiplicative interaction terms (GSD × age group and GSD × sex) to the multivariable logistic regression model. No significant interaction was observed (p for interaction for age = 0.687; p for interaction for sex = 0.577).

**Table 3:** Clinical Characteristics of the Longitudinal Cohort (N = 25,208)

Characteristics	Gallstones (N = 1,144)	No Gallstones (N = 24,064)	*p* value
**Demographic data**			
Age, yr	55±9	51±10	< 0.001
Men, n (%)	397 (35)	8,079 (34)	0.443
Body mass index, kg/m^2^	25±4	24±4	< 0.001
Waist circumference, cm	85±6	83±10	< 0.001
Smoke, ever, n (%)	285 (25)	5,570 (23)	0.173
Alcohol status, ever, n (%)	97 (9)	1,965 (8)	0.374
Physical activity, yes, n (%)	556 (49)	10,956 (46)	0.042
Education status ≥ college, n (%)	494 (43)	11,650 (48)	0.003
**Comorbidity**			
Hypertension, n (%)	217 (19)	2,844 (12)	< 0.001
Diabetes mellitus, n (%)	95 (8)	1,160 (5)	< 0.001
Dyslipidemia, n (%)	145 (13)	1,644 (7)	< 0.001
Gout, n (%)	50 (4)	820 (3)	0.078
Irritable bowel syndrome, n (%)	46 (4)	551 (2)	< 0.001
Chronic kidney disease, n (%)	24 (2)	320 (1)	0.036
**Laboratory data**			
Creatinine, mg/dL	0.7±0.2	0.7±0.3	0.526
Fasting Glucose, mg/dL	98±20	96±20	0.001
Total cholesterol, mg/dL	194±34	196±35	0.315
Triglyceride, mg/dL	118±64	113±83	0.007
Uric acid, mg/dL	5.6±1.4	5.4±1.4	< 0.001
**Follow-up, months**	46±13	47±14	0.026

Continuous variables are presented as mean ± standard deviation

**Table 4:** Association Between GSD and KSD: Incidence and Adjusted Hazard Ratios Stratified by Age and Sex (N = 25,208)

Variables	No. of kidney stone cases (%)	Number at risk	Unadjusted hazard ratio (95% CI)	*p* value	Adjusted hazard ratio (95% CI)*	*p* value**
**All**						
Gallstones, no	587 (2)	24,064	1.00 (Reference)	-	1.00 (Reference)	-
Gallstones, yes	55 (5)	1,144	2.14 (1.62 to 2.82)	< 0.001	1.801 (1.36 to 2.38)	< 0.001
**Age ≤ 50 years old**						
Gallstones, no	2,543 (4)	58,102	1.00 (Reference)	-	1.00 (Reference)	-
Gallstones, yes	124 (7)	1,668	2.22 (1.34 to 3.69)	0.002	2.06 (1.23 to 3.43)	0.006
**Age ≥ 50 years old**						
Gallstones, no	4,575 (8)	57,917	1.00 (Reference)	-	1.00 (Reference)	-
Gallstones, yes	492 (13)	3,863	1.92 (1.38 to 2.67)	< 0.001	1.73 (1.24 to 2.41)	0.001
**Men**						
Gallstones, no	332 (4)	8,079	1.00 (Reference)	-	1.00 (Reference)	-
Gallstones, yes	25 (6)	397	1.59 (1.06 to 2.39)	0.025	1.37 (0.91 to 2.07)	0.135
**Women**						
Gallstones, no	255 (2)	15,985	1.00 (Reference)	-	1.00 (Reference)	-
Gallstones, yes	30 (4)	747	2.80 (1.92 to 4.09)	< 0.001	2.44 (1.66 to 3.58)	< 0.001

GSD, gallstone disease; KSD, kidney stone disease; CI, confidence interval. *Multivariable analysis adjusted for age, sex, body mass index, waist circumference, smoking status, physical activity, educational status, presence of hypertension, diabetes mellitus, dyslipidemia, gout, irritable bowel syndrome, chronic kidney disease, serum creatinine, fasting glucose, triglycerides, and uric acid.** p for interaction was obtained by adding multiplicative interaction terms (GSD × age group and GSD × sex) to the multivariable Cox proportional hazards model (p for interaction for age = 0.416; p for interaction for sex = 0.043).
